# Expression of PD-1/PD-L1 axis in mediastinal lymph nodes and lung tissue of human and experimental lung fibrosis indicates a potential therapeutic target for idiopathic pulmonary fibrosis

**DOI:** 10.1186/s12931-023-02551-x

**Published:** 2023-11-14

**Authors:** Theodoros Karampitsakos, Apostolos Galaris, Serafeim Chrysikos, Ourania Papaioannou, Ioannis Vamvakaris, Ilianna Barbayianni, Paraskevi Kanellopoulou, Sofia Grammenoudi, Nektarios Anagnostopoulos, Grigoris Stratakos, Matthaios Katsaras, Fotios Sampsonas, Katerina Dimakou, Effrosyni D. Manali, Spyridon Papiris, Bochra Tourki, Brenda M Juan-Guardela, Petros Bakakos, Demosthenes Bouros, Jose D Herazo-Maya, Vassilis Aidinis, Argyris Tzouvelekis

**Affiliations:** 1 Department of Respiratory Medicine, University Hospital of Patras, Rio, Greece; 2 Ubben Center and Laboratory for Pulmonary Fibrosis Research, Morsani College of Medicine, University of South Florida, 33620 Tampa, FL USA; 3 Institute of Bio- Innovation, Biomedical Sciences Research Center Alexander Fleming, Athens, Greece; 4 5th Department of Pneumonology, Hospital for Thoracic Diseases, “SOTIRIA”, Athens, Greece; 5 Department of Pathology, Hospital for Thoracic Diseases, “SOTIRIA”, Athens, Greece; 6 First Academic Department of Pneumonology, “SOTIRIA”, Medical School, Hospital for Thoracic Diseases, National and Kapodistrian University of Athens, Athens, Greece; 7 2nd Pulmonary Medicine Department, Athens Medical School, “ATTIKON” University Hospital, National and Kapodistrian University of Athens, Athens, Greece

**Keywords:** Idiopathic pulmonary fibrosis, Mediastinal lymph nodes, Lung cancer, PD-1/PD-L1 axis, Pembrolizumab

## Abstract

**Background:**

Mediastinal lymph node enlargement is prevalent in patients with idiopathic pulmonary fibrosis (IPF). Studies investigating whether this phenomenon reflects specific immunologic activation are lacking.

**Methods:**

Programmed cell death-1 (PD-1)/ programmed cell death ligand-1 (PD-L1) expression in mediastinal lymph nodes and lung tissues was analyzed. PD-1, PD-L1 mRNA expression was measured in tracheobronchial lymph nodes of mice following bleomycin-induced injury on day 14. Finally, the effect of the PD-1 inhibitor, pembrolizumab, in bleomycin-induced pulmonary fibrosis was investigated.

**Results:**

We analyzed mediastinal lymph nodes of thirty-three patients (n = 33, IPF: n = 14, lung cancer: n = 10, concomitant IPF and lung cancer: n = 9) and lung tissues of two hundred nineteen patients (n = 219, IPF: 123, controls: 96). PD-1 expression was increased, while PD-L1 expression was decreased, in mediastinal lymph nodes of patients with IPF compared to lung cancer and in IPF lungs compared to control lungs. Tracheobronchial lymph nodes isolated on day 14 from bleomycin-treated mice exhibited increased size and higher PD-1, PD-L1 mRNA levels compared to saline-treated animals. Pembrolizumab blunted bleomycin-induced lung fibrosis, as indicated by reduction in Ashcroft score and improvement in respiratory mechanics.

**Conclusions:**

Mediastinal lymph nodes of patients with IPF exhibit differential expression profiles than those of patients with lung cancer indicating distinct immune-mediated pathways regulating fibrogenesis and carcinogenesis. PD-1 expression in mediastinal lymph nodes is in line with lung tissue expression. Lower doses of pembrolizumab might exert antifibrotic effects. Clinical trials aiming to endotype patients based on mediastinal lymph node profiling and accordingly implement targeted therapies such as PD-1 inhibitors are greatly anticipated.

**Supplementary Information:**

The online version contains supplementary material available at 10.1186/s12931-023-02551-x.

## Background

Mediastinal lymph node enlargement in high resolution computed tomography (HRCT) is prevalent in patients with idiopathic pulmonary fibrosis (IPF) and associated with worse clinical outcomes [1]. However, mediastinal lymphadenopathy of patients with IPF had been considered simply reactive in the past. Efforts to sample lymph nodes in these patients and investigate their immune profile have lagged behind. During the last years, several reports have demonstrated that a considerable proportion of patients with IPF (~ 10%) develop lung cancer with increasing incidence as survival is being prolonged [2]. Epidemiologic reports have fueled mechanistic discoveries showing that IPF and lung cancer share many common pathogenetic mechanisms as well as therapeutic compounds [3–6]. Whether enlarged mediastinal lymph nodes in IPF express cancer-related pathways remains to be addressed.

The hypothesis that mediastinal lymph node enlargement in IPF might reflect specific immunologic activation leading to disease progression deserves further investigation [7]. Investigation of cancer-associated pathways in mediastinal lymph nodes of patients with IPF may harbor important clinical implications. The programmed cell death-1 (PD-1)/ programmed cell death ligand-1 (PD-L1) pathway represents an adaptive immune resistance mechanism exerted by tumor cells in response to endogenous immune anti-tumor activity [8, 9]. PD-1 is a coinhibitory surface receptor mainly expressed on activated T cells, while it is also expressed on B cells, natural killer cells and myeloid-derived suppressor cells [10]. On the other hand, PD-L1 is the ligand of PD-1 and is expressed by antigen-presenting cells and tissue cells such as cancer cells. PD-L1 binds to its receptor, PD-1, in order to modulate either activation or inhibition [10]. The concept of blocking PD-1 and PD-L1 for the treatment of cancer has gained increasing attention and several inhibitors of the axis have been approved for the treatment of multiple types of cancer [8].

Recent studies implicated PD-1/PD-L1 axis in the pathogenesis of pulmonary fibrosis [11–15]. Upregulation of PD-1 in systemic CD4 + T cells promoted pulmonary fibrosis [11], while PD-L1 activation in fibroblasts of patients with IPF promoted invasion in vitro and lung fibrosis in vivo [12]. Membrane-bound PD-L1 was detected in alveolar and bronchiolar epithelial cells of the pulmonary parenchyma of a considerable proportion of patients with IPF [16]. However, to date there are no studies investigating the expression profiles of PD-1/PD-L1 in mediastinal lymph-node derived lymphocytes or the effects of PD-1 inhibitors such as pembrolizumab on pulmonary fibrosis. On the basis of the above, we aimed to investigate the expression of PD-1/PD-L1 axis and CD4/CD8 ratio in mediastinal lymph nodes of human and experimental lung fibrosis. We also sought to determine if mediastinal lymph node expression is in line with lung tissue expression. Finally, we investigated whether the PD-1 inhibitor, pembrolizumab, exerts anti-fibrotic properties in the bleomycin model of lung fibrosis.

## Methods

### Clinical part

In this multicenter, prospective study, we included three groups of patients with evidence of mediastinal lymphadenopathy on HRCT (1. IPF, 2. IPF and non-small cell lung cancer, 3. Non-small cell lung cancer) between 19/1/2018 and 16/5/2020. Exclusion criteria were: (1) presence of emphysema, (2) prior treatment for IPF or lung cancer and (3) refusal or inability to provide informed consent. Protocol, data collection and analysis were approved by the Institutional Review Board and the Local Ethics Committee (protocol number: 38/18-01-2018). IPF was diagnosed based on ATS/ERS/JRS/ALAT Guidelines 2018 [17]. All patients provided written informed consent to undergo endobronchial ultrasound-guided transbronchial needle aspiration (EBUS-TBNA). EBUS-TBNA was performed at the time point of diagnosis. More details for the EBUS-TBNA procedure and the pathologic evaluation are provided in the online supplement.

#### Gene expression data in lung tissue

We aimed to show that expression in mediastinal lymph nodes is in accordance with lung tissue. Therefore, we analyzed gene expression data from the Lung Genomics Research Consortium (LGRC) cohort data set. Data are available at GSE47460 as well as in the website of LGRC [http://www.lung-genomics.org/) and have been described in [18]. In particular, we compared lung tissue expression of PD-1 and PD-L1 between patients with IPF and control subjects. Control subjects had normal lung histology.

#### Outcome measures

Primary outcome in the clinical part was the expression of PD-1/PD-L1 axis and CD4/CD8 ratio in mediastinal lymph nodes of patients with IPF compared to patients with lung cancer. Secondary outcome was the gene expression of PD-1/PD-L1 in IPF lungs and control lungs.

### Experimental part

For more details, see the online supplement (Supplemental Figs. 1 and 2).

#### Animals

All studies in mice, in line with the ARRIVE guidelines, have been approved by the Veterinary service and Fishery Department of the local governmental prefecture (#278,202 and #986,221), following the positive opinion of the Institutional Protocol Evaluation Committee of BSRC Alexander Fleming.

Pulmonary fibrosis was induced through the administration of 0.8U/Kg of bleomycin (Nippon Kayaku) to anesthetized mice (IP ketamine/xylazine/atropine, 100/10/0.05 mg/kg, respectively) via the oropharyngeal (OA) route, as it has been previously described [19]. All mice were 8–10 weeks old and the group characteristic details are described in every experiment separately. Sample size was decided using power analysis based on a previously published report from our lab [19].

#### Lymph node dissection and Flow Cytometry analysis

Tracheobronchial lymph nodes were isolated from the animals during euthanization on day 14 and immediately placed in ice. Diameter > 2 mm was considered abnormal based on existing literature [20]; yet, given that our approach was novel and there are limited data for the normal size compared to humans, we compared the actual lymph node size. Six mice per group were used for the lymph node analysis and comparison. The dissection was performed by flushing the tissue through a 40 μm cell strainer with RPMI-1640 (11875-093, Gibco). The samples were centrifuged at 1.200 g for 10 min at 4^o^C. The supernatant was discarded and the cell pellet was resuspended in blocking solution (Supplemental Table 1). After 15 min of incubation in ice, fresh 3% BSA PBS was added and the samples were centrifuged at 1.200 g for 7 min at 4^o^C. The supernatant was removed, the conjugated antibodies were added (Supplemental Table 1) and the cells were placed in ice for 30 min in dark. Then fresh 3% BSA PBS was added and the samples were centrifuged at 1.200 g for 7 min at 4^o^C. Finally, the supernatant was removed, the stained cells were resuspended in 250µL of filtered PBS and data were acquired on a BD FACSCantoTM II flow cytometer using the BD FACSDiva software (BD Biosciences). The analysis of raw data was performed using the Flowjo v10.8.1 software.

**Fig. 1:**
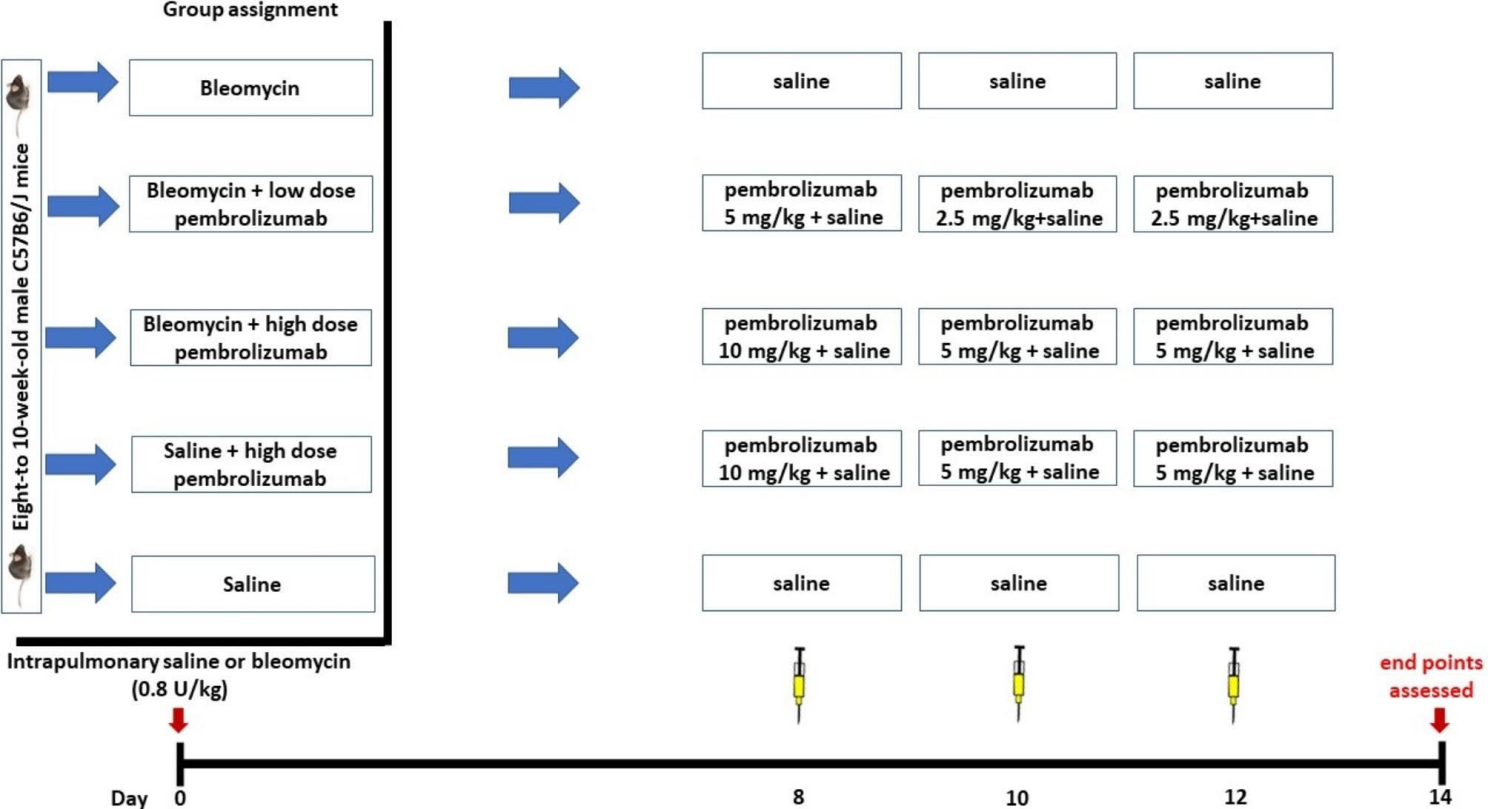
Schematic representation of pembrolizumab therapeutic protocol. Eight-to-10 week old male C57B6/J mice were randomly assigned in the following groups: (1) saline, (2) saline + high dose pembrolizumab, (3) bleomycin, (4) bleomycin + low dose pembrolizumab, (5) bleomycin + high dose pembrolizumab **(**Fig. 1**)**. Pembrolizumab (A2005, Selleckchem) was diluted in saline as recommended by the supplier at a final concentration of 1.5 mg/mL and was injected intraperitoneally on days 8, 10 and 12 following bleomycin administration (Day 0). High dose pembrolizumab stands for 10 mg/kg, 5 mg/kg and 5 mg/kg on days 8, 10 and 12, respectively. Low dose pembrolizumab stands for 5 mg/kg, 2.5 mg/kg and 2.5 mg/kg on days 8, 10 and 12, respectively. On day 14, Ashcroft score, body weight, total protein, cellularity and respiratory mechanics were compared among the aforementioned groups. Nine mice were initially used for each arm (total: 9 mice for 5 arms-45 mice)

#### Pembrolizumab therapeutic protocol

**Fig. 2:**
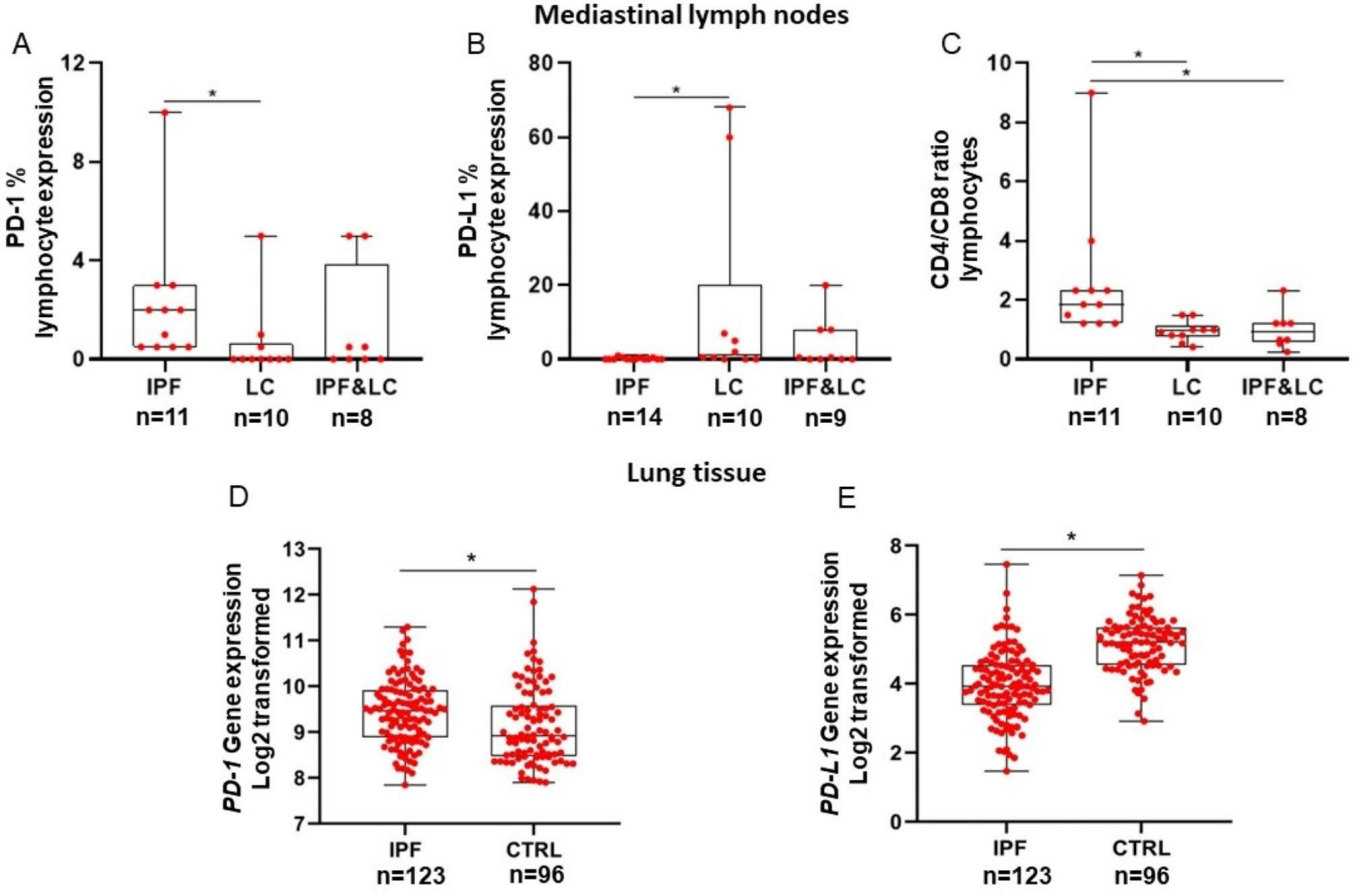
Mediastinal lymph nodes: Median PD-1% expression was significantly higher in lymphocytes of patients with IPF compared to lung cancer (IPF: 2.0, 95% CI: 0.5 to 3.0 vs. lung cancer: 0.0, 95% CI: 0.0 to 0.8, Kruskal–Wallis test; p = 0.02), **(Panel A).** Median PD-L1% expression was significantly lower in lymphocytes of patients with IPF compared to lung cancer (IPF: 0.0, 95% CI: 0.0 to 0.5 vs. lung cancer: 1.3, 95% CI: 0.0 to 34.8, Kruskal–Wallis test; p = 0.04), **(Panel B).** Median CD4/CD8 ratio was significantly higher in mediastinal lymph nodes of patients with IPF compared to patients with lung cancer and patients with concomitant IPF/lung cancer (IPF: 1.9, 95% CI: 1.2 to 2.6, vs. lung cancer: 0.9, 95% CI: 0.7 to 1.3, vs. IPF and lung cancer: 0.9, 95% CI: 0.5 to 1.4, Kruskal–Wallis test; p = 0.001), **(Panel C).** Lung tissue: Analysis of the Lung Genomics Research Consortium cohort showed significantly increased expression of PD-1 and decreased expression of PD-L1 in patients with IPF (n = 123) compared to control (CTRL) subjects with normal lung histology (n = 96), (PD-1: IPF: 9.5, 95% CI: 9.3 to 9.6, vs. controls: 8.9, 95% CI: 8.8 to 9.3, Mann- Whitney test; p = 0.001), (PD-L1: IPF: 4.0 ± 1 vs. controls: 5.1 ± 0.8, Unpaired t test; p < 0.0001) **(Panel D and E)**

Eight-to-10 week old male C57B6/J mice were randomly assigned in the following groups: (1) saline, (2) saline + high dose pembrolizumab, (3) bleomycin, (4) bleomycin + low dose pembrolizumab, (5) bleomycin + high dose pembrolizumab **(**Fig. 2**)**. Pembrolizumab (A2005, Selleckchem) was diluted in saline as recommended by the supplier at a final concentration of 1.5 mg/mL and was injected intraperitoneally on days 8, 10 and 12 following bleomycin administration (Day 0). High dose pembrolizumab stands for 10 mg/kg, 5 mg/kg and 5 mg/kg on days 8, 10 and 12, respectively. The loading dose of 10 mg/kg was based on previous experimental studies [21]. Low dose pembrolizumab stands for 5 mg/kg, 2.5 mg/kg and 2.5 mg/kg on days 8, 10 and 12, respectively. On day 14, Ashcroft score, body weight, total protein, cellularity and respiratory mechanics were compared among the aforementioned groups. Nine mice were initially used for each arm (total: 9 mice for 5 arms-45 mice). AG was responsible for the allocation.

#### Outcome measures

Primary outcome in the experimental part was the effect of PD-1 inhibition in bleomycin treated mice. Secondary outcomes included: (1) the expression of PD-1/PD-L1 axis and CD4/CD8 in tracheobronchial lymph nodes of bleomycin-treated mice compared to saline treated-mice, (2) comparison of the size of tracheobronchial lymph nodes from bleomycin-treated mice and saline treated-mice.

### Statistical analysis

Summary descriptive statistics were generated with categorical data displayed as absolute numbers and relative frequencies. Continuous data were denoted as mean ± standard deviation (SD) or medians with 95% Confidence Interval (95% CI) following Kolmogorov-Smirnov test for normality. Mann Whitney U test or t-test was used for the investigation of significant differences based on the absence or presence of normality in each case. Kruskal-Wallis test was performed to detect differences in case more than two groups were compared. p-values < 0.05 were considered statistically significant.

## Results

### Clinical part

We analyzed PD-1, PD-L1 expression and CD4/CD8 ratio in mediastinal lymph nodes of thirty-three patients (n = 33) (IPF: 14, IPF and lung cancer: 9, lung cancer: 10). In four patients (IPF:3, IPF and lung cancer:1), only PD-L1 was analyzed due to adequacy of the sample. With regards to gene expression of *PD-1* and *PD-L1* in lung tissue, we included in the analysis one hundred twenty-three IPF lungs (n = 123) and ninety-six control lungs (n = 96) from the LGRC cohort. Patient demographics and disease characteristics are summarized in Table 1.

**Table 1:** Baseline characteristics of patients included in the analysis

Characteristics	IPF	IPF and lung cancer	Lung cancer	IPF	Controls
**Sample**	Mediastinal lymph nodes	Mediastinal lymph nodes	Mediastinal lymph nodes	Lung tissue	Lung tissue
**Number of patients** **Mean age ± SD** **Male** **Female** **Current smokers** **Ex smokers** **Never smokers** **FVC% predicted ± SD** **DLCO% predicted ± SD**	14 71.4 ± 8.3 13 (92.9%) 1 (7.1%) 1 (7.1%) 11 (78.6%) 2 (14.3%) 86.5 ± 14.9 57.6 ± 22.6	9 69.2 ± 5.7 8 (88.9%) 1 (11.1%) 3 (33.3%) 6 (66.7%) 0 (0.0%) 77.6 ± 12.9 48.3 ± 17.4	10 67.3 ± 6.6 8 (80%) 2 (20%) 2 (20%) 7 (70%) 1 (10%) 79.2 ± 3.2 58.5 ± 9.3	123 64.8 ± 8.3 82 (66.7%) 41 (33.3%) 1 (0.8%) 76 (61.8%) 46 (37.4%) 64.6 ± 16.4 48.8 ± 18.0	96 63.9 ± 11.2 46 (47.9%) 50 (52.1%) 8 (8.3%) 48 (50.0%) 40 (41.7%) 94.8 ± 13.3 82.8 ± 17.0

### Increased CD4/CD8 ratio, PD-1 expression and decreased PD-L1 expression was observed in lymphocytes derived from mediastinal lymph nodes of patients with IPF compared to lung cancer

**Fig. 3:**
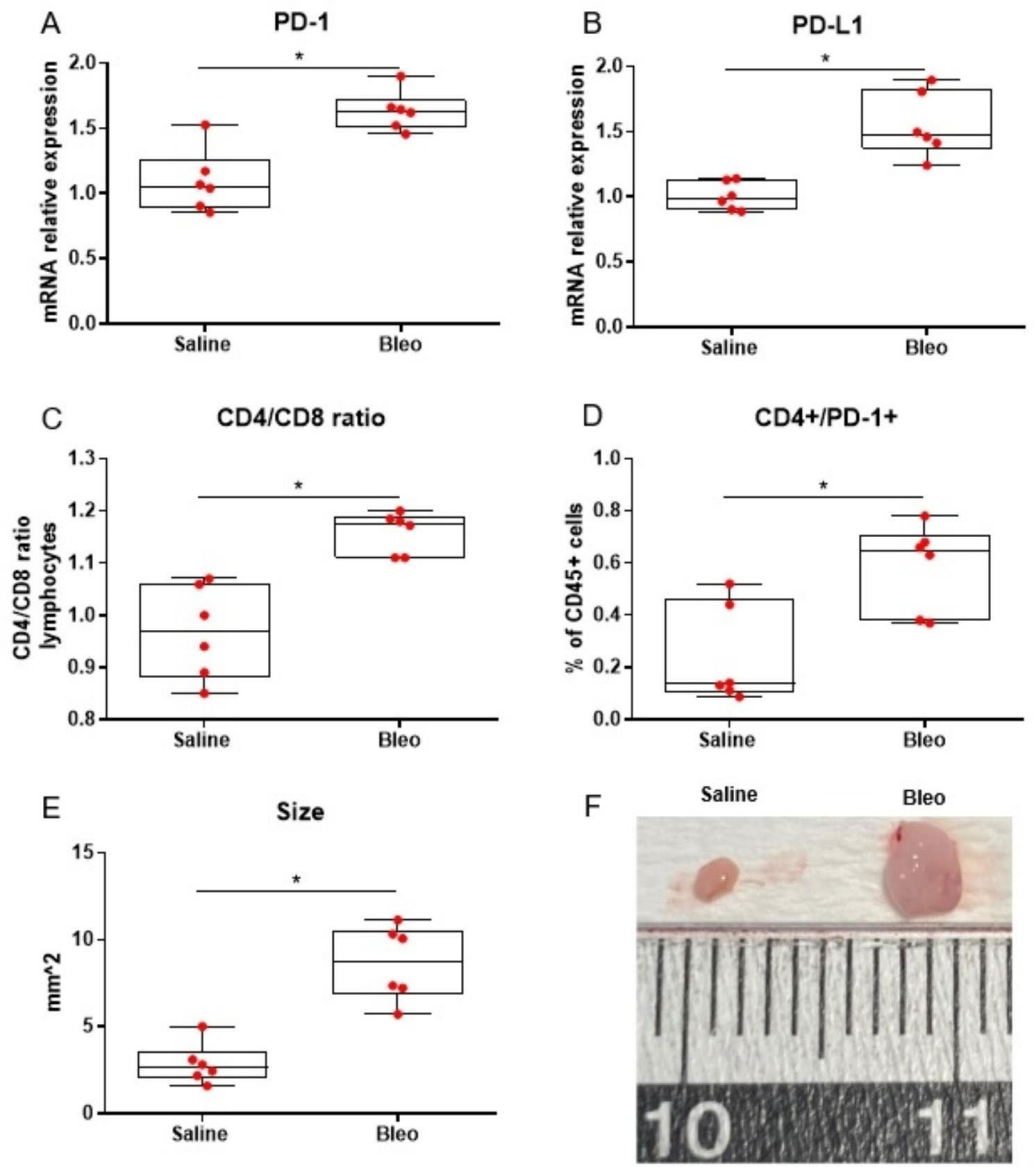
Median PD-1 mRNA expression was significantly higher in lymph nodes isolated from mice treated with bleomycin compared to littermates treated with saline at day 14 (Mann-Whitney test; p = 0.009), **(Panel A).** Median PD-L1 mRNA expression was significantly higher in lymph nodes isolated from mice treated with bleomycin compared to littermates treated with saline at day 14 (Mann-Whitney test; p = 0.002), **(Panel B).** Median CD4/CD8 ratio was significantly higher in mediastinal lymph nodes isolated from mice treated with bleomycin compared to littermates treated with saline at day 14 (Mann-Whitney test; p = 0.002), **(Panel C).** Median CD4+/PD-1 + double positive cells percentage out of CD45 + cells was significantly higher in mediastinal lymph nodes isolated from mice treated with bleomycin compared to littermates treated with saline at day 14 (Mann-Whitney test; p = 0.026), **(Panel D).** The size (mm^2^) of lymph nodes isolated from mice treated with bleomycin was significantly increased compared to saline-treated mice at day 14 (Mann-Whitney test; p = 0.002), **(Panel E).** Representative images of lymph nodes isolated from mice treated with bleomycin and littermates treated with saline at day 14 (**Panel F)**. Six mice were used in each arm (two independent experiment of three mice per arm, each, total mice: 12)

Median PD-1% expression was significantly higher in lymphocytes derived from mediastinal lymph nodes of patients with IPF compared to lung cancer (IPF: 2.0, 95% CI: 0.5 to 3.0 vs. lung cancer: 0.0, 95% CI: 0.0 to 0.8, p = 0.02), **(**Fig. 3, **Panel A)**. Median PD-L1% expression was significantly lower in lymphocytes of patients with IPF compared to lung cancer (IPF: 0.0, 95% CI: 0.0 to 0.5 vs. lung cancer: 1.3, 95% CI: 0.0 to 34.8, p = 0.04), **(**Fig. 3, **Panel B)**. Median CD4/CD8 ratio was significantly higher in mediastinal lymph nodes of patients with IPF compared to patients with lung cancer and patients with concomitant IPF/lung cancer (IPF: 1.9, 95% CI: 1.2 to 2.6, vs. lung cancer: 0.9, 95% CI: 0.7 to 1.3, vs. IPF and lung cancer: 0.9, 95% CI: 0.5 to 1.4, p = 0.001), **(**Fig. 3, **Panel C)**. There were no significant differences in the expression of PD-1/PD-L1 axis and CD4/CD8 between patients with lung cancer and patients with concomitant IPF and lung cancer. Representative images are presented in Supplemental Fig. 3.

### PD-1 is increased and PD-L1 is decreased in IPF lungs

Analysis of LGRC gene expression data demonstrated that PD-1 was significantly increased in IPF lungs (n = 123) compared to control lungs (n = 96) (IPF: 9.5, 95% CI: 9.3 to 9.6, vs. controls: 8.9, 95% CI: 8.8 to 9.3, p = 0.001), **(**Fig. 3, **Panel D)**. PD-L1 was significantly decreased in IPF lungs compared to control lungs (IPF: 4.0 ± 1 vs. controls: 5.1 ± 0.8, p < 0.0001), **(**Fig. 3, **Panel E)**.

### Experimental part

#### Tracheobronchial lymph nodes from bleomycin-treated mice exhibit increased PD-1, PD-L1 mRNA levels and CD4/CD8 ratio

Tracheobronchial lymph nodes isolated on day 14 from bleomycin-treated mice exhibited increased PD-1 (1.63 fold, p = 0.009) and PD-L1 (1.55 fold, p = 0.002) mRNA levels compared to saline-treated animals, **(**Fig. 1, **Panel A and B)**. CD4/CD8 ratio was higher in CD45 + cells isolated on day 14 from tracheobronchial lymph nodes of bleomycin-treated mice compared to saline-treated mice (1.18, 95% CI: 1.11 to 1.20, vs. 0.97, 95% CI: 0.86 to 1.07, p = 0.002), **(**Fig. 1, **Panel C)**. Increased CD4+/PD-1 + double positive cells (%of CD45 + cells) isolated on day 14 from tracheobronchial lymph nodes of bleomycin-treated mice compared to saline-treated littermates were observed (p = 0.026), **(**Fig. 1, **Panel D)**. Bleomycin-treated mice on day 14 had significantly larger tracheobronchial lymph nodes (mm^2^, short axis x long axis) compared to saline-treated littermates (10.73, 95% CI: 7.77 to 12.80, vs. 2.62, 95% CI: 1.69 to 5.37, p = 0.002), **(**Fig. 3, **Panel E and F)**.

### Pembrolizumab exerts anti-fibrotic properties

**Fig. 4:**
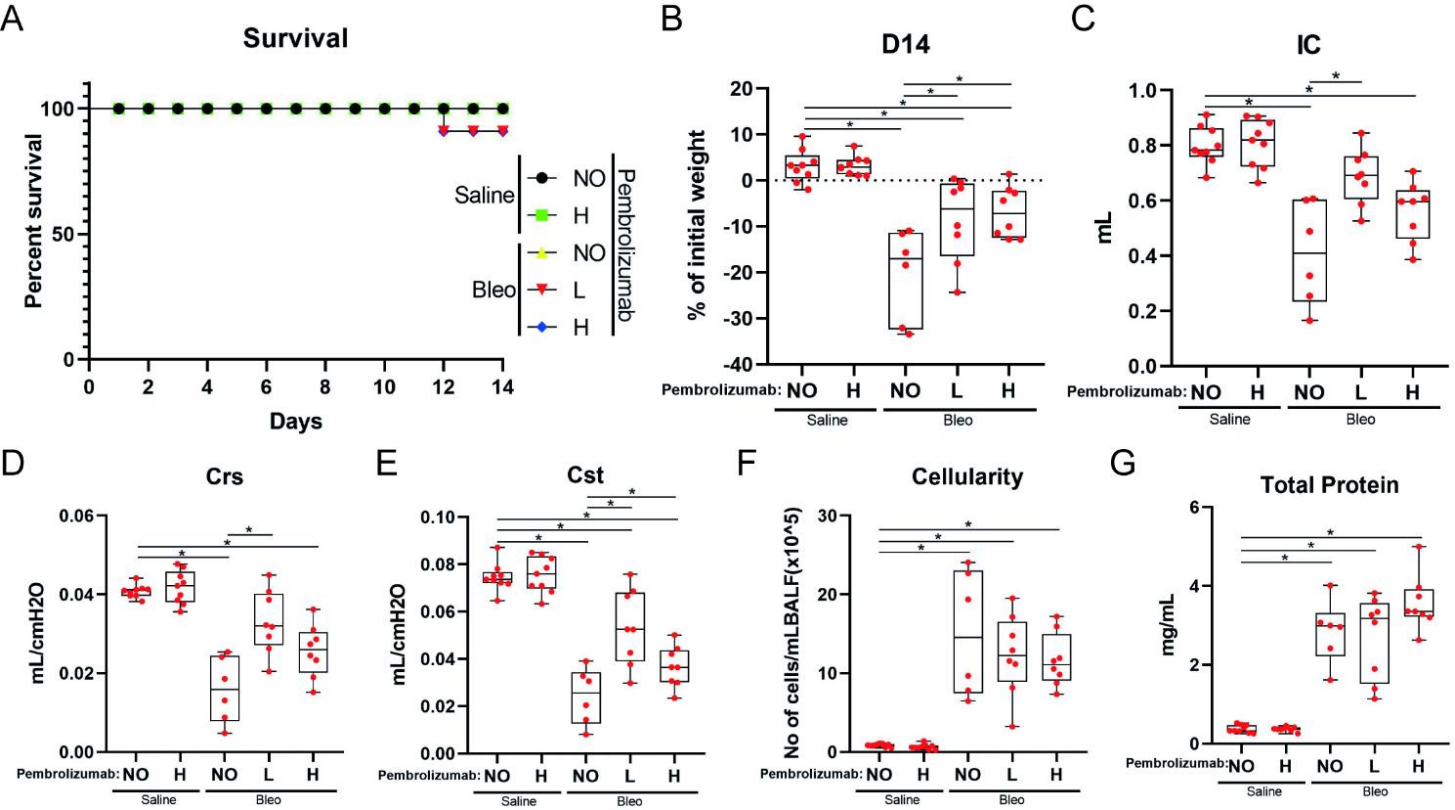
Parameters evaluated following the implementation of pembrolizumab therapeutic protocol. NO, L and H stand for no pembrolizumab, low-dose pembrolizumab and high-dose pembrolizumab, respectively. Cst, Crs and IC stand for static compliance, dynamic compliance and inspiratory capacity, respectively. Survival plot of mice included in the study. Two mice (low dose pembrolizumab: 1, high dose pembrolizumab: 1) were euthanized due to humane endpoints guidelines on day 12 **(Panel A)**. All bleomycin-treated groups had statistically significant lower body weight than saline groups. Mice treated both with pembrolizumab and bleomycin had statistically significant higher weight compared to bleomycin-treated mice (Kruskal–Wallis test; bleomycin vs bleomycin and low dose pembrolizumab: p = 0.013, bleomycin vs bleomycin and high dose pembrolizumab: p = 0.004) **(Panel B)**. Bleomycin-treated mice had statistically significant lower IC compared to mice treated with low dose pembrolizumab and bleomycin (Kruskal–Wallis test;p < 0.001) **(Panel C)**. Bleomycin-treated mice had significantly lower Crs compared to both pembrolizumab groups (Kruskal–Wallis test; bleomycin vs bleomycin and low dose pembrolizumab: p < 0.001, bleomycin vs bleomycin and high dose pembrolizumab: p = 0.035) **(Panel D)**. Bleomycin-treated mice had significantly lower Cst compared to mice treated with low dose pembrolizumab and bleomycin (Kruskal–Wallis test; p < 0.001). High dose pembrolizumab led to statistically significant lower Cst compared to low dose pembrolizumab (Kruskal–Wallis test; p = 0.025) **(Panel E)**. BALF cellularity of all bleomycin-treated groups was significantly higher compared to the saline treated groups (Kruskal–Wallis test; p < 0.001 for every comparison) **(Panel F)**. Total protein concentration in BALF was significantly higher in all bleomycin-treated groups compared to the saline treated groups (Kruskal–Wallis test; p < 0.001 for every comparison). There was a trend for higher total protein concentration in the ‘’high dose’’ compared to the ‘’low dose’’ pembrolizumab group (Kruskal–Wallis test; p = 0.07) **(Panel G)**

Two mice were euthanized due to humane endpoints guidelines on day 12, as they had almost lost 25% of their body weight **(**Fig. 4, **Panel A)**. Bleomycin-treated mice exhibited significantly lower body weight compared to saline-treated mice (p < 0.0001). Pembrolizumab reduced weight loss (bleomycin vs bleomycin and low dose pembrolizumab: p = 0.013, bleomycin vs bleomycin and high dose pembrolizumab: p = 0.004) **(**Fig. 4, **Panel B)**. Mice treated with bleomycin and low dose pembrolizumab presented with improved respiratory mechanics as indicated by: increased inspiratory capacity (p < 0.001), dynamic lung compliance (p < 0.0001) and static lung compliance (p < 0.0001), compared to bleomycin-treated mice. Mice treated with high dose pembrolizumab also exhibited improved dynamic lung compliance compared to bleomycin-treated mice (p = 0.035), **(**Fig. 4, **Panel C, D and E)**. With regards to bronchoalveolar lavage fluid (BALF), there was no significant difference in the total cell count among groups **(**Fig. 4, **Panel F)**, while there was a trend for higher total protein concentration in the ‘’high dose’’ compared to the ‘’low dose’’ pembrolizumab group (p = 0.07) **(**Fig. 4, **Panel G).**

**Fig. 5:**
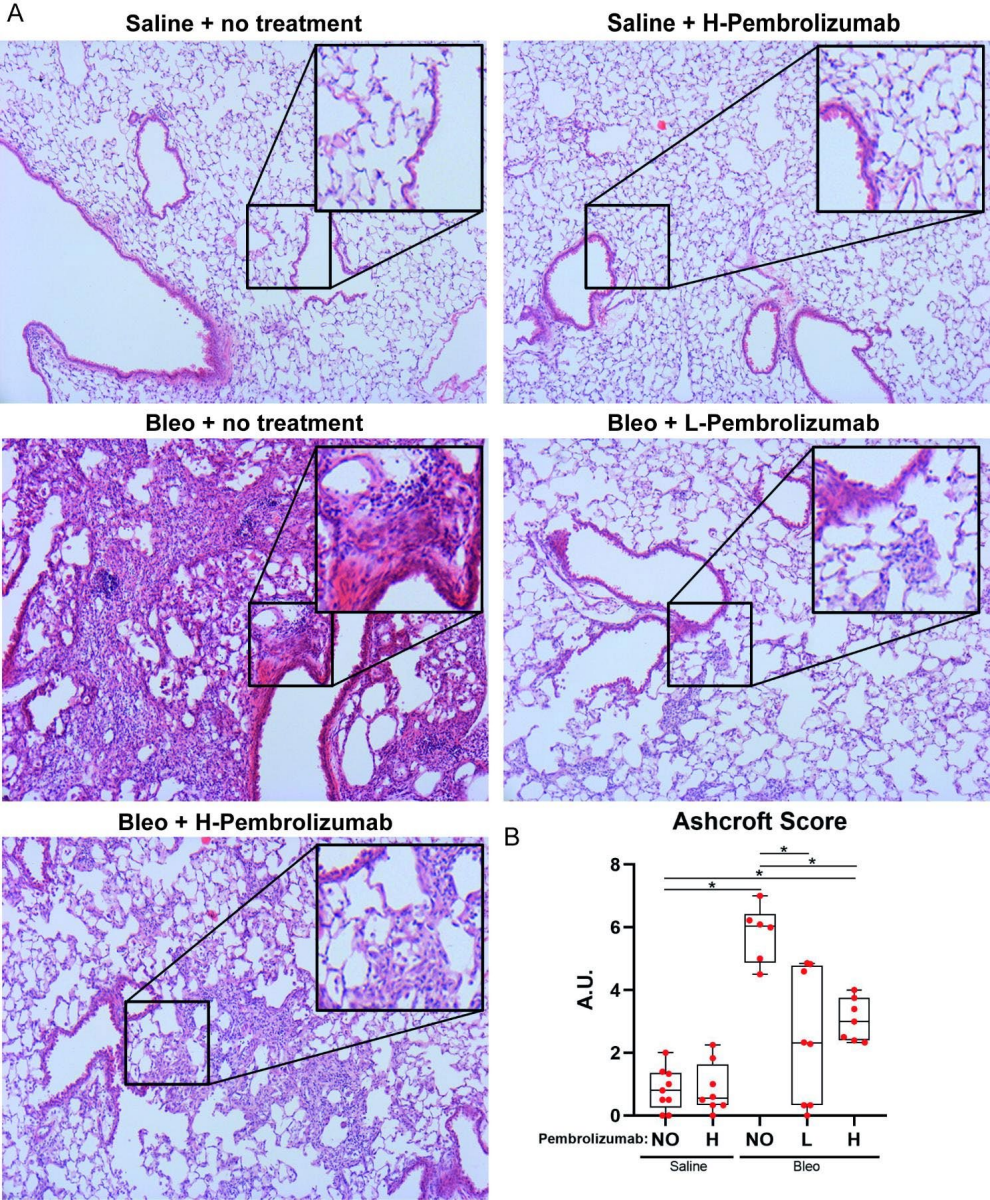
Representative images of Hematoxylin & Eosin (H&E) staining of lung tissue from every group indicating less fibrosis severity in pembrolizumab treated animals **(Panel A)**. NO, L and H stand for no pembrolizumab, low-dose pembrolizumab and high-dose pembrolizumab, respectively. Ashcroft score for all groups estimated by H&E images. Bleomycin-treated mice and mice treated with bleomycin and high dose pembrolizumab had significantly higher Ashcroft score compared to saline-treated mice (Kruskal–Wallis test; saline vs. bleomycin: p < 0.001, saline vs. high dose pembrolizumab: p = 0.006). Mice treated with bleomycin and either low or high dose pembrolizumab had significantly lower Ashcroft score compared to bleomycin-treated mice (Kruskal–Wallis test; bleomycin vs. bleomycin and low dose pembrolizumab: p < 0.001, bleomycin vs. bleomycin and high dose pembrolizumab: p = 0.002) **(Panel B)**

Pembrolizumab reduced fibrotic changes as indicated by semiquantitative Ashcroft score **(**Fig. 5**)**. Representative images of lung tissue from every group indicated no fibrosis in the saline treated group, severe fibrosis in bleomycin control group and significantly lower fibrosis in pembrolizumab-treated mice **(**Fig. 5, **Panel A)**. Both low and high dose pembrolizumab led to significant reduction in Ashcroft score compared to treatment with bleomycin alone (bleomycin vs. bleomycin and low dose pembrolizumab: p < 0.0001, bleomycin vs. bleomycin and high dose pembrolizumab: p = 0.002), **(**Fig. 5, **Panel B)**.

**Fig. 6:**
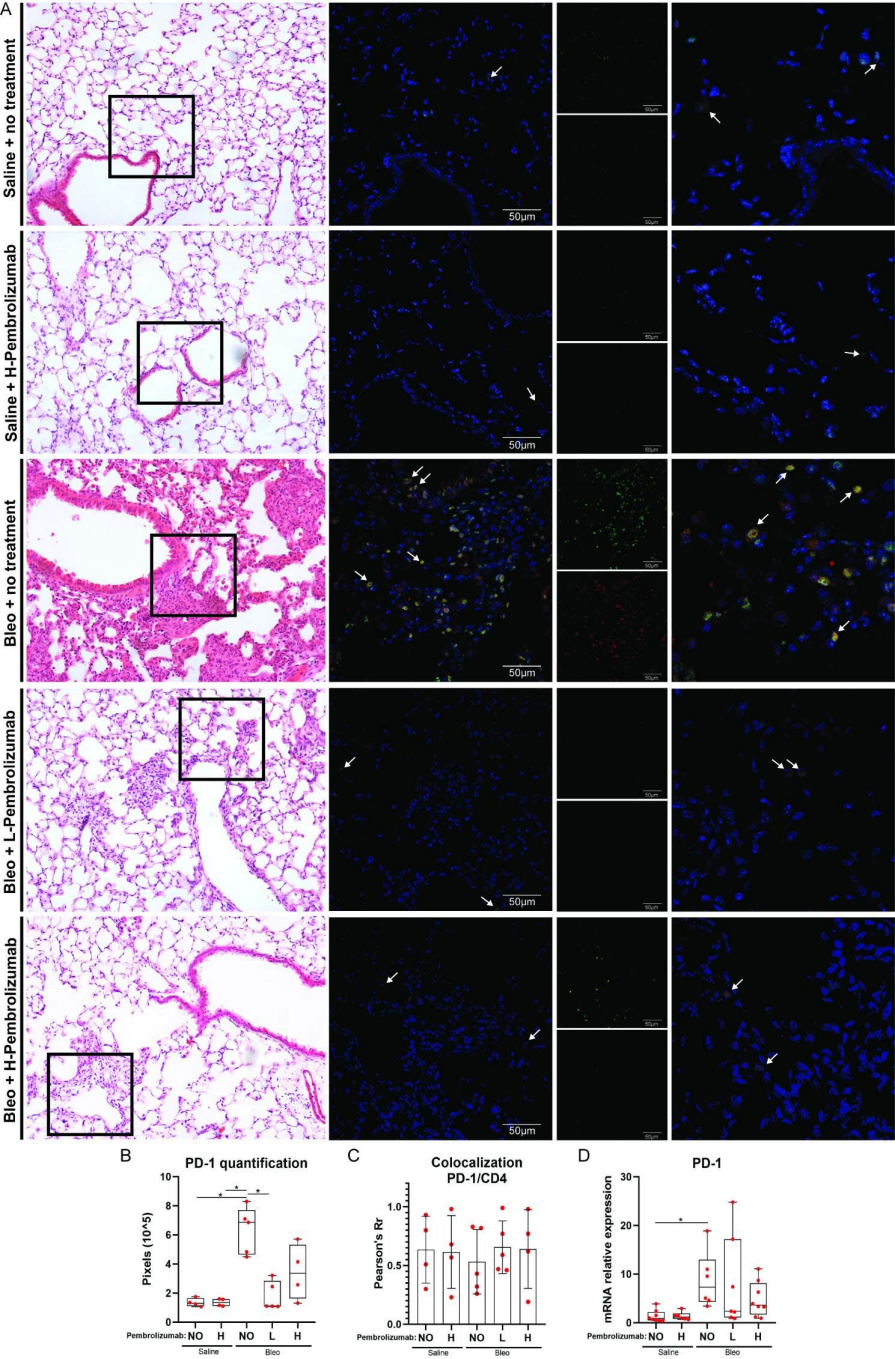
H&E images (left column) showing exact sections (black boxes) that were double stained against PD-1 and CD4. PD-1 and CD4 positivity is demarcated with green and red respectively, while DAPI which was used to stain the nuclei is demarcated with blue. Double stain images indicated higher PD-1 and CD4 expression upon bleomycin administration. Right column shows 2X zoomed images. White arrows are pointing representative double positive cells. (error bars: 50 μm) **(Panel A)**. Quantification analysis showed that bleomycin treated mice presented with higher PD-1 compared to saline treated mice (p = 0.04), while mice treated with bleomycin and low dose pembrolizumab presented with lower PD-1 compared to mice treated with bleomycin only (p = 0.03, Kruskal-Wallis test) **(Panel B)**. Pearson’s Rr quantification measurement of the colocalization of PD-1 and CD4. Treatment with pembrolizumab and/or bleomycin did not result to a significant change in the percentage of PD-1/CD4 colocalization **(Panel C)**. Median PD-1 mRNA expression was significantly higher in lung tissues isolated from mice treated with bleomycin compared to littermates treated with saline at day 14 (Kruskal–Wallis test; saline vs. bleomycin: p = 0.008). Pembrolizumab treatment did not lead to statistically significant change in PD-1 mRNA expression **(Panel D)**

Finally, double immunofluorescent staining revealed increased PD-1 and CD4 lung expression in bleomycin-treated mice compared to saline-treated mice **(**Fig. 6, **Panel A)**. Quantification analysis showed that bleomycin treated mice presented with higher PD-1 compared to saline treated mice (p = 0.04), while mice treated with bleomycin and low dose pembrolizumab presented with lower PD-1 compared to mice treated with bleomycin only (p = 0.03, Kruskal-Wallis test) **(**Fig. 6, **Panel B)**. PD-1 was highly colocalized with CD4. Pembrolizumab did not alter significantly the percentage of PD-1/CD4 colocalization **(**Fig. 6, **Panel C)**. Median PD-1 mRNA expression was significantly higher in lung tissues isolated from mice treated with bleomycin compared to littermates treated with saline at day 14(Kruskal–Wallis test; saline vs. bleomycin: p = 0.008). Pembrolizumab administration did not lead to statistically significant change in PD-1 lung mRNA expression **(**Fig. 6, **Panel D)**.

## Discussion

This is the first study providing evidence that mediastinal lymph node enlargement reflects immunologic activation in patients with IPF. Following the observation that PD-1 was consistently increased in mediastinal lymph nodes of patients with IPF and tracheobronchial lymph nodes of bleomycin-treated mice compared to control groups, we studied the role of the PD-1 inhibitor, pembrolizumab, in experimental pulmonary fibrosis in mice. Pembrolizumab exerted anti-fibrotic properties, as indicated by reduction in Ashcroft score and improvement in respiratory mechanics. PD-1 + CD4 + cells could represent potential therapeutic targets for lung fibrosis.

Mediastinal lymph nodes of patients with IPF had higher PD-1 and lower PD-L1 lymphocyte expression compared to patients with lung cancer. Given the need (1) to compare IPF and controls, which was not feasible through EBUS and (2) to determine whether mediastinal lymph node expression is in accordance with lung tissue expression, we analyzed gene expression data of the LGRC cohort. In accordance with expression in mediastinal lymph nodes, gene expression analysis of the LGRC cohort showed that PD-1 was increased and PD-L1 was decreased in IPF lungs compared to control lungs. Previous studies investigating independently lung tissue expression of PD-1 or PD-L1 have reported increased expression of both compared to controls [11, 14, 15, 22]. The differential expression of PD-1 and PD-L1 that we found has already been demonstrated in oncogene-addicted non-small cell lung cancer [23]. In a cohort of patients with adenocarcinoma, patients expressing PD-1 were more frequently male, smokers, while patients expressing PD-L1 were more frequently female, never or former smokers [23]. It remains to be addressed whether disease severity has a role in the expression, as well. To this end, the aforementioned provide rationale for the differential expression of PD-1 and PD-L1.

Mediastinal lymph nodes of patients with IPF exhibited increased CD4/CD8 ratio compared to patients with lung cancer. Our results are in line with previous evidence demonstrating higher proportion of CD4 + than CD8 + cells in the BALF of patients with IPF [24]. These data revive the interest for the role of immunity in the pathogenesis of IPF [25–27]. Importantly, despite the fact that CD4 + T cells are increased as a total in IPF, it seems that diverse subsets present with diverse functions and thus different CD4 + T subpopulations can exert opposing pro- and anti-fibrotic properties [25, 28–30]. Of note, transcriptional profiling of peripheral blood mononuclear cells showed that underexpression of T cell regulatory genes linked to lower percentages of CD4 + CD28 + T cells was associated with reduced transplant free survival [31]. Marked suppression of functional CD4+, CD25high, FoxP3 + cells in the peripheral blood and BALF of patients with IPF has also been reported [32]. Most recently, two distinct subsets of lung resident CD4 + T cells have been identified during chronic inflammation, a pro-fibrotic CD103^lo^ and an anti-fibrotic CD103^hi^ population [33].

To this end, we aimed to investigate the expression of PD-1/PD-L1 axis and CD4/CD8 ratio in the bleomycin model of pulmonary fibrosis. Our clinical data were further corroborated by experimental evidence showing similar expression patterns in the bleomycin model of lung fibrosis including increased PD-1, PD-L1 mRNA levels and CD4/CD8 ratio in tracheobronchial lymph nodes compared to saline-treated mice. Of note, CD4+/PD-1 + cells isolated on day 14 from tracheobronchial lymph nodes of bleomycin-treated mice were significantly increased compared to saline-treated mice. The aforementioned finding couples with previous elegant, experimental evidence showing that PD-1 up-regulation on CD4 + T cells promoted lung fibrosis through STAT3-mediated IL-17 A and TGF-b1 production [11]. Multiple other studies have shown increased lung tissue expression of PD-1 and PD-L1 in different experimental models of lung fibrosis [14, 15, 22, 34–37]. A key finding for PD-L1 was its association with fibroblasts’ phenotype [12, 14]. The most representative paradigm of that is the evidence that PD-L1 on invasive fibroblasts drove fibrosis in a humanized mice model of IPF [12].

Following the observation that PD-1 was upregulated in human and experimental lung fibrosis, we showed that low dose pembrolizumab exerted anti-fibrotic properties, as indicated by reduction in Ashcroft score and improvement in respiratory mechanics. In line with our findings, an elegant study reported that blockade of PD-1 pathway significantly altered expression of IL-17 A in CD4 + T cells mediated through pSTAT3 downregulation, leading thus to significant declines in collagen-1 production [11]. PD-1/PD-L1 inhibition seemed to alleviate silica-induced lung fibrosis, as well [34]. In our study, Pembrolizumab did not alter the percentage of PD-1/CD4 colocalization, while it reduced PD-1 in the quantification analysis of immunofluorescence. Pembrolizumab did not significantly reduce PD-1 mRNA expression, despite there was a trend for reduction in the high dose arm. Of note, previous evidence has suggested that pembrolizumab mainly acts through blocking the engagement between PD-1 and its ligands instead of reducing PD-1 expression per se [38]. Disruption of PD-1/PD-L1 interaction might be the key to limit pulmonary fibrosis progression [37, 39].

The concept that lower doses of pembrolizumab might have a beneficial role for patients with fibrotic lung diseases deserves further investigation. Implementation of lower doses of PD-1 inhibitors might be efficacious in IPF patients with specific immune profile, while concomitantly spare adverse events of PD-1 inhibitors such as pneumonitis [40–42]. Given the emerging role of PD-L1 inhibitors including durvalumab and PD-1 inhibitors such as pembrolizumab in the treatment of non-small cell lung cancer, clinicians will often confront the dilemma of their use in patients with IPF and concomitant lung cancer [43–49]. A substantial proportion of patients receiving the approved dose of PD-1 inhibitors develops pneumonitis and the risk is higher for patients with IPF [49, 50]. Thus, clinical trials aiming to investigate the effect of low dose pembrolizumab in patients with fibrotic lung diseases through drug repurposing seem rational. A potential trial in IPF should take into consideration that the first phase 3 clinical trials of pembrolizumab leading to US Food and Drug Administration approval for cancer used a 2 mg/kg dosage every 3 weeks [51]. The fixed dosage of 200 mg every 3 weeks was subsequently established given that it presented with pharmacokinetic equivalence and the dosage of 2 mg/kg was logistically challenging [50]. However, this difference might be crucial for patients with IPF who are at high risk for pneumonitis. A lower dose of 150 mg should be adequate for patients with IPF and weight of approximately 75 kg [51]. Besides, nintedanib was seminally introduced for the treatment of refractory lung adenocarcinoma and was then approved in lower doses for the treatment of IPF [52].

Taken together, our study exhibited a number of important attributes and provided novel insights with potentially important clinical implications. First, PD-1+/CD4 + T cells might be a future therapeutic target in IPF. Targeted therapies for patients with the appropriate endotype might not have the deleterious effects of previously used immunomodulatory compounds inhibiting non-specifically T-lymphocytes. Towards this direction, we showed that low dose pembrolizumab exerted anti-fibrotic properties. Secondly, our study highlights the role of mediastinal lymphadenopathy in IPF. Expression in mediastinal lymph nodes was in line with lung tissue. Given that (1) ideally we need to sample lung tissue (where the disease is) for endotyping, but this approach has lagged due to complications, (2) expression in mediastinal lymph nodes was in line with lung tissue and (3) EBUS seems to be a safer procedure than BAL and lung biopsy in experienced centers [53], the concept of endotyping patients with IPF through EBUS might hold promise. A study investigating on a longitudinal basis the risk for lung cancer development based on the endotyping of mediastinal lymph nodes would also be of particular interest. In the not so distant far, assays of CD4 T-cells derived from mediastinal lymph nodes might identify patients with IPF at greater risk for progression or lung cancer development and patients more likely to experience benefit from targeted immunotherapy.

On the other hand, our study presents with some limitations. Other immunologic or non-immunologic contributors to pulmonary fibrosis were not assessed experimentally. Thus, we cannot present a complete mechanism able to facilitate progression of fibrotic lung disease in humans and murine models. The effect of pembrolizumab in specific cell types was not investigated. Expression of PD-1 has been described in multiple cell types including T-cells, while PD-L1 cellular source varies and includes monocytes and macrophages [54–56]. With regards to IPF, publicly available single cell data show expression of PD-1 mainly in CD4/CD8 T cells and of PD-L1 in macrophages [56]. Based on that, PD-1 inhibition might block the interaction of PD-1 positive T cells with PD-L1 positive macrophages, similarly with the block of the interaction of PD-1 positive T cells and PD-L1 positive tumor cells in cancer. Future studies investigating this hypothesis are greatly anticipated. Further investigation of T-cell exhaustion and myeloid derived suppressor cells in pulmonary fibrosis also holds promise. With regards to the analysis of EBUS-TBNA results in the clinical part, the sample size was not large, while in a few patients only PD-L1 was analyzed due to sample adequacy. However, the sample size seems to be acceptable taking into consideration especially the prevalence of cases with concomitant IPF and lung cancer. Moreover, we could not report long-term follow-up data for patients with IPF based on their PD-1/PD-L1 profile; yet, the goal of this work was to fuel future studies aiming to implement targeted therapies such as PD-1 inhibitors to patients with the appropriate endotype [6], as indicated by mediastinal lymph node profiling.

## Conclusion

This is the first study providing direct evidence in favor of the concept that mediastinal lymphadenopathy in IPF is not simply reactive. PD-1 and PD-L1 expression in mediastinal lymph nodes was in line with lung tissue expression. Mediastinal lymph nodes of patients with IPF had significantly higher PD-1 expression and CD4/CD8 ratio compared to patients with lung cancer, indicating distinct immune-mediated pathways regulating fibrogenesis and carcinogenesis. Tracheobronchial lymph nodes from bleomycin-treated mice considerably enlarged and exhibited increased PD-1, PD-L1 mRNA levels and increased CD4/CD8 ratio compared to saline-treated animals. Pembrolizumab exerted anti-fibrotic properties in the bleomycin model of pulmonary fibrosis. The concept of endotyping patients with IPF through sampling of mediastinal lymph nodes instead of lung tissue and implementing accordingly targeted therapies such as PD-1 inhibitors deserves further investigation.

### Electronic supplementary material

Below is the link to the electronic supplementary material.

Supplementary Material 1

Supplementary Material 2

Supplementary Material 3

Supplementary Material 4

## Data Availability

Data are available upon reasonable request to the corresponding author.
