# Supplementary material for: Expression of PD-1/PD-L1 axis in mediastinal lymph nodes and lung tissue of human and experimental lung fibrosis indicates a potential therapeutic target for idiopathic pulmonary fibrosis
Source: Respir Res. 2023 Nov 14;24:279. doi: 10.1186/s12931-023-02551-x (PMC10648728; doi:10.1186/s12931-023-02551-x)
Supplement: Supplementary file 1 — Supplementary Material 1 [file 12931_2023_2551_MOESM1_ESM.docx]

**Expression of PD-1/PD-L1 axis** **in mediastinal lymph nodes and lung tissue of human and experimental lung fibrosis** **indicates a potential therapeutic target for Idiopathic Pulmonary Fibrosis**

Theodoros Karampitsakos^1,2*^, Apostolos Galaris^3*^, Serafeim Chrysikos^4^, Ourania Papaioannou^1^, Ioannis Vamvakaris^5^, Ilianna Barbayianni^3^, Paraskevi Kanellopoulou^3^, Sofia Grammenoudi^3^, Nektarios Anagnostopoulos^6^, Grigoris Stratakos^6^, Matthaios Katsaras^1^, Fotios Sampsonas^1^, Katerina Dimakou^4^, Effrosyni D. Manali^7^, Spyridon Papiris^7^, Bochra Tourki^2^, Brenda M Juan-Guardela^2^, Petros Bakakos^6^, Demosthenes Bouros^6^, Jose D Herazo-Maya^2^, Vassilis Aidinis^3#^, Argyris Tzouvelekis^1#^

*equally contributed to this work, # jointly supervised that work

^1^ Department of Respiratory Medicine, University Hospital of Patras, Greece, ^2^ Ubben Center and Laboratory for Pulmonary Fibrosis Research, Morsani College of Medicine, University of South Florida, Tampa, FL 33620, USA , ^3^ Institute of Bio-Innovation, Biomedical Sciences Research Center Alexander Fleming – Athens, Greece, ^4^ 5th Department of Pneumonology, Hospital for Thoracic Diseases, “SOTIRIA”, Athens, Greece, ^5^Department of Pathology, Hospital for Thoracic Diseases, “SOTIRIA”, Athens, Greece, ^6^ First Academic Department of Pneumonology, Hospital for Thoracic Diseases, “SOTIRIA”, Medical School, National and Kapodistrian University of Athens, Athens, Greece, ^7^ 2nd Pulmonary Medicine Department, "ATTIKON" University Hospital, Athens Medical School, National and Kapodistrian University of Athens, Greece

Correspondence to:

Argyrios Tzouvelekis MD, MSc, PhD

Associate Professor of Respiratory Medicine

Head Department of Respiratory Medicine

University of Patras, Greece

[argyris.tzouvelekis@gmail.com](mailto:argyris.tzouvelekis@gmail.com)

**Supplementary Materials & Methods**

Clinical part

EBUS TBNA

We implemented an ‘’all-comers design’’ during the aforementioned period. All patients with 1. IPF, 2. IPF and lung cancer that fulfilled the criteria entered the study. Patients with lung cancer were enrolled in the study using a 1:1 ratio between them and patients with concomitant IPF/lung cancer. In particular, following the inclusion of the first patient with lung cancer, we included one patient with lung cancer right after the inclusion of a new patient with IPF/lung cancer. In the lung cancer arm, 50% of patients (n=5/10) had adenocarcinoma, 40% (n=4/10) squamous cell lung cancer and 10% (n=1/10) not-otherwise specified non-small cell lung cancer.

Lymph nodes of 2R, 2L, 3, 4R, 4L, 5, 6, and 7 stations were reviewed and mediastinal lymphadenopathy/enlargement was defined as a node with a short-axis diameter of 10 mm or more. EBUS-TBNA (convex probe-EBUS, BF-UC 180F; Olympus;Tokyo;Japan) was performed concomitantly with conventional bronchoscopy. Prior EBUS-TBNA, anesthesia with spr.Lidocain 10% and Midazolam/Fentanyl were applied at the discretion of clinicians. Biopsy was performed with 22 Gauge Olympus needles. Tissue was stabilized in a 10% formalin solution and incubated with hydrogen peroxide.

Pathologic evaluation of mediastinal lymph nodes

Formalin-fixed paraffin embedded tissue was sectioned at 5μ m, deparaffinized, rehydrated with antigen retrieval being performed with PT Link, Dako in envision flex target retrieval solution (high pH, 1x working solution and incubation for 20 minutes at 97 °C). With regards to PD-L1 only, antigen retrieval was performed with Envision Flex Target Retrieval Solution in a different setting (low pH cat no K.8005, 1x for 20minutes at 97 °C).

The following monoclonal antibodies were used for pathologic evaluation of the tissue obtained: i) CD4 clone SP35, Cell marque, catNo104R-14(California USA)1/100diluition, 30 minutes incubation at room temperature ii) CD8 clone C8/144B, Dako-Agilent (St CLARA USA), cat no M7103,1/250 diluition, 30 minutes incubation at room temperature iii) PD-1 clone NAT 105, RTU, Ready-to-use, BioSB(St Barbara USA),CAT NO E214,RTU 40 minutes incubation at room temperature iv) PD-L1 clone 22C3, Dako-Agilent(St CLARA USA) CAT NO M3653 ,1/50diluition, 30 minutes incubation at room temperature.

Immunohistochemistry was performed in an AUTOSTAINER LINK 48 Dako-Agilent Denmark according to manufacturer’s protocol (EnvisionTM FLEX, High pH, (Link), Dako-Agilent Denmark). Pathologist’s report included % lymphocyte expression of PD-1, PD-L1 and CD4/CD8 ratio for each sample. In four cases, only PD-L1 was analyzed due to adequacy of the sample and given that PD-L1 expression is the first provided in clinical practice.

Experimental part

Animals

All studies in mice, in line with the ARRIVE guidelines, have been approved by the Veterinary service and Fishery Department of the local governmental prefecture (#278202 and #986221), following the positive opinion of the Institutional Protocol Evaluation Committee of BSRC Alexander Fleming. All animals were bred under specific-pathogen-free conditions at the local animal facility of BSRC “Alexander Fleming” at “20–22◦C, 55% ± 5 humidity and a 12-h light/dark cycle. Water and food were given “ad libitum”.

Respiratory Mechanics examination by Flexi-Vent system-SCIREQ

We used the Flexi-Vent SCIREQ computer-controlled piston ventilator system to evaluate the effect of pembrolizumab in respiratory mechanics. According to the manufacturer’s protocol, mice were anesthetized with ketamine. Subsequently, mice were tracheotomized and ventilated using the Flexi-Vent system. Average breathing frequency was 150 breaths/min. Briefly, pulmonary parenchyma was inflated during maximal vital capacity (MVC) perturbation to a standard pressure of +30 cm H_2_O and subsequently was deflated to determine MVC. Maximal pressure–volume loops between total lung capacity (+30 cm H_2_O) and functional residual capacity (2.8 cm H_2_0) were finally generated to obtain the static compliance (Cst) of the respiratory system. Dynamic compliance (Crs) was obtained through a single forced oscillation technique. Through the aforementioned forced oscillation technique and pressure-volume loop perturbation, inspiratory capacity (IC) was also recorded. Each manoeuver was repeated until three acceptable results (coefficient of determination above 0.95 was needed) were obtained in every mouse. The average of the three acceptable results was measured.

Euthanization protocol and sample collection

Euthanization of all animals was performed in a gradually filled CO2 chamber at the timepoints mentioned in every model. BALF was obtained by lavaging the airways with 3mL of normal saline using a cannula through the trachea (three times; 1mL each). Then, BALF was centrifuged for 15 minutes at 1.200g at 4^o^C. The first 1mL of the BALF was transferred without the cells into a new siliconized tube. The other 2mLs were discarded; the cells were pooled and treated with GEYS solution for 10 minutes in ice. Then they were centrifuged for 10 minutes at 1.200g at 4^o^C, the suspension was discarded, the cell pellet was resuspended in fresh PBS and the cells were counted under an inverted microscope using a Newbauer chamber. The lungs were filled with formalin (143091.1214, AppliChem) in order to be later mounted into paraffin. Additionally, total protein concentration was estimated in the BALF using bradford reagent (Cat.no.: 39222.03, SERVA) following the manufacturer’s instructions. Standard Curve was constructed using known concentrations of Bovine Serum Albumin (Cat.no: A-7030, Sigma).

RNA extraction and real-time-PCR

Tracheobronchial lymph nodes were isolated from the animals during euthanization and were immediately transferred into liquid nitrogen. Lymph nodes were homogenized in 1mL of Trizol (TR118, Molecular Research Center) and subsequently total RNA extraction was performed according to manufacturer’s instructions. cDNA was constructed by reverse transcription using M-MLV reverse transcriptase (28025-013, Invitrogen) according to manufacturer’s instructions. Real-time polymerase chain reaction (RT-PCR) was performed using SoFAst EvaGreen Supermix on a Bio-Rad CFX96 Touch™ Real-Time PCR Detection System (Bio-Rad Laboratories Ltd, CA, USA) and the values were normalized to β2-microglobulin (B2M). The primers used were: B2M (F: 5´-TTCTGGTGCTTGTCTCACTGA-3´; R: 5´-CAGTATGTTCGGCTTCCCATTC-3´), PD-1 (F: 5’-CAAGGACGACACTCTGAAGGA-3’; R: 5’-GTCCAGCTCCTCATAGGCCA-3’) and PD-L1 (F: 5’-ACTGTGAAAGTCAATGCCCC-3’; R: 5’-TCCAGATGACTTCGGCCTTG-3’).

Immunohistochemistry/Immunofluorescence

Fixed lung tissues were mounted into paraffin and 4μm slices were cut and placed on slides. Then, hematoxylin/ eosin staining was performed according to common protocol. Immunofluorescence double staining against PD-1 and CD4 was performed on 4μm lung slices placed on superfrost slices. Briefly, lung sections were deparaffinized in xylene, rehydrated in a gradient of ethanol, and washed with water. Then, antigen retrieval with sodium citrate buffer pH 6.0 was performed by autoclave for 20min. Next, they were treated with blocking solution (10% normal goat serum in PBS-T) at room temperature for 1 h and incubated O/N with conjugated primary antibodies (PD-1: Biolegend, 135213; CD4: eBioscience, 56-0042-82). Following this, sections were washed 3 times with PBS-T and mounted with medium containing DAPI (Sigma, F6057) for nuclear visualization. Imaging was performed using a Nikon Eclipse E800 microscope (Nikon Corp., Shinagawa-ku, Japan) attached to a Q Imaging EXI Aqua digital camera, using the Q-Capture Pro 7 software. Colocalization quantification was performed by the “Colocalization Finder” plugin of the ImageJ software.

**Table 1:** Antibodies used in flow cytometry analysis.

| **Target** | **Fluorochrome** | **Dilution** | **Cat. No. (Manufacturer)** |
| --- | --- | --- | --- |
| CD45 | Alexa700 | 1:400 | 103128 (Biologend) |
| CD4 | APC-Cy7 | 1:400 | A15384 (Lifetech) |
| CD8 | Alexa 647 | 1:400 | 100727 (Biolegend) |
| PD-1 | FITC | 1:400 | 135213 (Biolegend) |
| PD-L1 | PE/Cyanine 7 | 1:400 | 124313 (Biolegend) |
| CD16/CD32 | Blocking | 1:400 | 101302 (Biolegend) |

**Supplemental Figure 1.** Cellularity and total protein in BALF of saline and bleomycin treated mice denoting the successful experiment.

**Supplemental Figure 2.** Gating strategy for the analysis of cell populations. In detail, firstly debris were excluded and then only single cells were gated. Live cells were gated as PI negative subjects and then only CD45+ cells were chosen for further analysis. Subsequently, CD4 and CD8 populations were discriminated and further analyzed for PD-1 and PD-L1 expression.

**Supplemental Figure 3.** Representative images for positive PD-1 and CD4/CD8 stainings (brown demarcation) of a patient with IPF, as well as a patient with IPF and concomitant lung cancer. Of note, PD-L1 staining is positive in 80% of cancer cells, but not in lymphocytes.
