# Supplementary figures and images for: Expression of PD-1/PD-L1 axis in mediastinal lymph nodes and lung tissue of human and experimental lung fibrosis indicates a potential therapeutic target for idiopathic pulmonary fibrosis
Source: Respir Res. 2023 Nov 14;24:279. doi: 10.1186/s12931-023-02551-x (PMC10648728; doi:10.1186/s12931-023-02551-x)

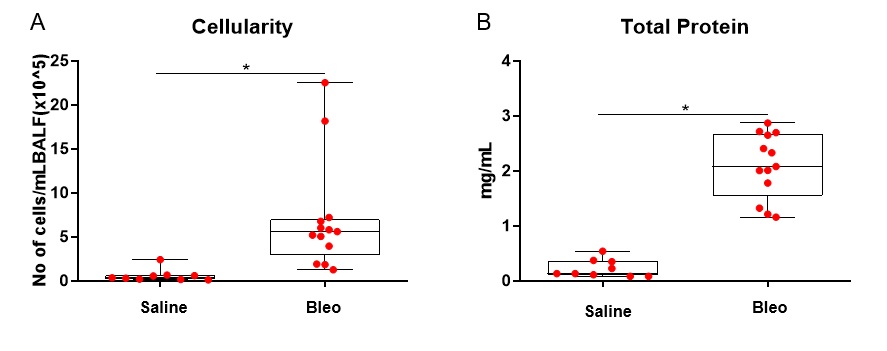

Supplement: Supplementary file 2 — Supplementary Material 2 [file 12931_2023_2551_MOESM2_ESM.jpg]

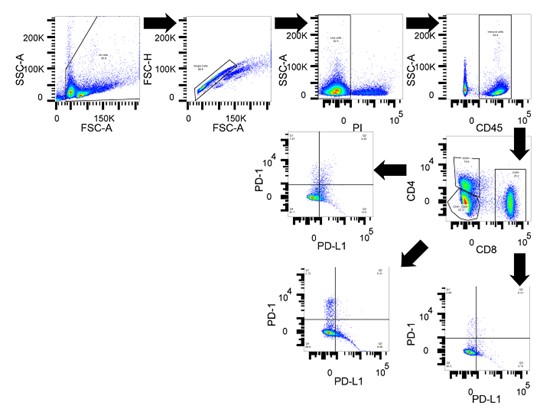

Supplement: Supplementary file 3 — Supplementary Material 3 [file 12931_2023_2551_MOESM3_ESM.jpg]

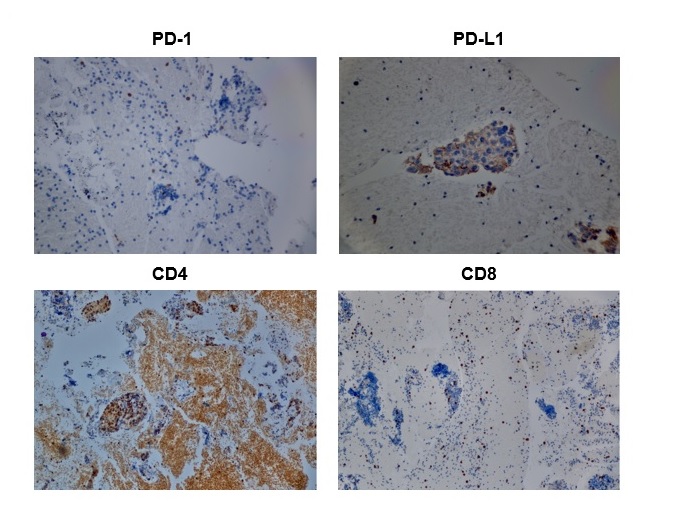

Supplement: Supplementary file 4 — Supplementary Material 4 [file 12931_2023_2551_MOESM4_ESM.jpg]
